# Wendy L. Havran, PhD: 1955–2020

**DOI:** 10.3390/cells9041039

**Published:** 2020-04-22

**Authors:** Deborah A. Witherden

**Affiliations:** Department of Immunology and Microbiology, The Scripps Research Institute, 10550 North Torrey Pines Rd, La Jolla, CA 92037, USA; deborahw@scripps.edu; Tel.: +1-858-784-8619

Wendy Havran, Professor and Associate Dean of Graduate Studies at Scripps Research, passed away on January 20th, 2020 following a heart attack. She was 64 years old. She will be sorely missed by family, friends, and colleagues throughout the world and will be forever remembered for her kindness and generosity of spirit.

Wendy was a world leader in the field of γδ T cell biology. Much of what we know about γδ T cells today is either directly or indirectly a result of Wendy’s pioneering work. Wendy was born in Houston, Texas on 1 September, 1955. A graduate of Duke University, Wendy completed her PhD in Frank Fitch’s lab at the University of Chicago. She then went on to do her postdoctoral studies with Jim Allison at the University of California, Berkeley. It was here that Wendy made the discovery that would shape the rest of her prolific academic career. Wendy identified a unique population of invariant γδ T cells that reside in the epidermal layer of the murine skin. Termed dendritic epidermal T cells, or DETC, for their highly dendritic morphology, these cells became the main focus of Wendy’s research for the next 30 years.

Wendy received the prestigious Lucille P. Markey Scholarship in 1989 and moved to the Scripps Research Institute in 1991, where she established her own research group. At Scripps, Wendy’s research characterized DETC function in depth and further discovered similar populations in human skin. Through these and subsequent studies, Wendy described the seminal role these epithelial-resident γδ T cells play during homeostasis and in tissue repair. Wendy went on to characterize the molecular interactions driving these repair processes and, more recently, turned her focus to the role these cells play in cancer. Throughout her career, Wendy’s work was always carefully done, thorough in nature, logical in manner, and so very detailed that it was published in some of the most prestigious journals.

Her calm, but firm, leadership style made her the “go-to” person for all manner of programs and committees. She was Associate Dean of the Scripps graduate program, established and ran national and international conferences, served on the editorial board of prominent journals, and was a member of numerous committees for the American Association of Immunologists. She was a leader who was not afraid to say, “we can do it!” She inspired her colleagues at Scripps to walk laps for the Leukemia and Lymphoma Society every year, pick up sandwiches and cookies for the International γδ T Cell Conference, and drive to all parts of southern California to deliver seminars for educational purposes. This is why she was honored by Scripps Research for mentorship, by the Leukemia and Lymphoma Society for fundraising, and by the American Association of Immunologists for distinguished service. Wendy was extremely humble in her receipt of these awards and much more enjoyed celebrating the achievements of others, particularly her students and postdocs. She would organize celebratory lunches or cakes and never missed an opportunity to let others know of each and every accomplishment of her lab family. Recently, the moment she was most proud of was watching her postdoc mentor, Jim Allison, receive the Nobel Prize. 

One of Wendy’s passions was helping and promoting women in science, both inside and outside of her lab. She had 40 female scientists work in her lab throughout her time at Scripps and so many of these women have gone on to leadership roles in academia, industry, and health care. Wendy always gave the trainees in her lab the scope to pursue an interest or passion outside the lab, whether it be a particular sport or hobby, or raising a family, without having to sacrifice their scientific careers. There was a time when four of the women in Wendy’s lab were pregnant at the same time. Instead of lamenting her unlucky fate, she celebrated at conferences stating that her lab had been very productive that year—and she was not talking about the science. Her openness to a flexible work schedule allowed her trainees to thrive and to learn to be effective at both time and people management.

There was a moment on the day after Wendy passed away that we were standing in her lab as students, faculty, and staff came by one after the other to talk about Wendy. All looking for solace. The solace we found was in happy stories about her taming wild cats, mentoring each of us, faculty/students/staff, and remembering friendly chats in her office. Some of the faculty were wondering how they were going to be the mentor that Wendy was, saying that, before giving advice, they would have to ask themselves, “what would Wendy say?” 

The γδ T cell field has lost a pioneer, a brilliant experimentalist, and an inspirational leader, and we have all lost an extraordinary colleague and friend.

